# Ideal memcapacitors and meminductors are overunity devices

**DOI:** 10.1038/s41598-020-73833-3

**Published:** 2020-10-07

**Authors:** Dimitri Jeltsema, Arjan van der Schaft

**Affiliations:** 1 School of Engineering and Automotive, HAN University of Applied Science, P.O. Box 2217, 6802 CE Arnhem, The Netherlands; 2 Bernoulli Institute for Mathematics, Computer Science and AI, University of Groningen, P.O. Box 407, 9700 AK Groningen, The Netherlands

**Keywords:** Engineering, Physics

## Abstract

It is rigorously proved that ideal memcapacitors and meminductors are *not* passive or cyclo-passive devices. Equivalently, this implies that there exist excitation profiles that allow to extract more energy from the device than it is supplied with; so that their energy conversion efficiency exceeds 100%. This means that ideal memcapacitors and meminductors violate the First Law of thermodynamics, and thus are non-physical as they constitute so-called *overunity* systems. An illustrative mechanical analogue is provided for which such an excitation profile is explicitly constructed. Hence ideal memcapacitors and meminductors are mathematical artefacts, and the question arises what this implies for the properties of non-ideal memcapacitors and meminductors (or, memcapacitive systems and meminductive systems), which do satisfy the First Law.

## Introduction

Batteryless circuit elements that are able to store information would represent a serious paradigm change in electronics, allowing for low-power computation and storage. One such candidate circuit element is the ideal *memristor*^1^, which gained a worldwide attention of both researchers and the mainstream media over the past decade since its discovery was announced^2^. The notion of memelements can also be extended to ideal capacitive and inductive devices, coined as *memcapacitors* and *meminductors*^3^. Generalizations to include any class of two-terminal devices whose resistance, capacitance, and inductance depends on the internal state of the system are known as memristive, memcapacitive, and meminductive systems (memsystems for short), respectively. Both ideal memelements and generalized memsystems have specific properties that typically appear most strikingly as a pinched hysteretic loop in the two constitutive variables that define them: current versus voltage for memristive systems, voltage versus charge for memcapacitive systems, and current versus flux-linkage for the meminductive systems.

Many real-world systems are argued to belong to the class of memsystems, especially those of nanoscale dimensions^3–5^. However, to the best of our knowledge, and apart from the fact that the real-world existence of an ideal memristor is critically addressed^6–8^, physical devices that unambiguously resemble an ideal memcapacitor or an ideal meminductor are yet to be found.

It is well-known that physical devices generally cannot release more energy than they are supplied with. This means that physical devices are *cyclo-passive* systems. At most they can be locally active, in the sense that at some interval within a given time-frame of a cyclic motion energy might be released but will need to be supplied at another interval within the same time-frame.

From a historical perspective, the mathematical description of an ideal memcapacitor and meminductor dates back to the early eighties in the context of so-called higher-order elements^9–11^. In these works, the dissipativity analysis (which was at that time relatively new) is unfortunately erroneously applied and led to the conclusion that ideal memcapacitors and meminductors are cyclo-passive (in fact, *lossless*) under the condition that the memcapacitance or meminductance is non-negative. This has perhaps misguided several researchers to believe that ideal memcapacitors and meminductors are relevant (as ideal device models) for as long as the non-negativity condition is fulfilled.

Recently, starting from a microscopic level, doubts have been raised about the non-negativity condition and the realizability of memelements in the context of Kubo’s response theory^12^. Our approach is somewhat complementary and starts from a thermodynamical perspective that rigorously proves that ideal memcapacitors and meminductors are neither passive nor cyclo-passive. Devices that are not cyclo-passive are able to produce energy in a repeatable manner, and are known as so-called *overunity* systems or, in a more classical parlance, *perpetual motion* systems. Hence, the invention or discovery of real-world energy-storing memelements which are close to ideal memelements also becomes more unlikely.

The remainder of the paper is organized as follows. First, the mathematical essentials of ideal memcapacitors and meminductors are collected in “Energy-storing memelements”. “Dissipativity and cyclo-dissipativity theory” briefly recalls the theory of dissipativity and cyclo-dissipativity, which is instrumental in “Ideal energy-storing memelements are not cyclo-passive” to analyze the (cyclo-)passivity properties of the ideal memcapacitor and meminductor. The outcome of the analysis is exemplified in “Overunity devices?” by using a recently proposed mechanical analogue. Some concluding remarks are provided in “Concluding remarks”.

## Energy-storing memelements

Let *V* and *I* denote the port voltage and current, respectively, and their respective time-integrals, $\varphi$ and *q*, the flux-linkage and charge, or, equivalently, $\dot{\varphi } = V$ and $\dot{q} = I$.

### Memcapacitor

An ideal memcapacitor^3^ is defined by a constitutive relationship between flux-linkage $\varphi$ and time-integrated charge $\rho$, i.e.,

1 $$\begin{aligned} \rho = \widehat{\rho }(\varphi ) \ {\text {or}} \ \varphi = \widehat{\varphi }(\rho ). \end{aligned}$$

Differentiating the latter with respect to time yields the following dynamical systems.

• Voltage-controlled memcapacitor:

2 $$\begin{aligned} \dot{\varphi }&= \frac{q}{C(\varphi )}, \nonumber \\ \dot{q}&= I \quad \text {(input)},\nonumber \\ \text {(output)} \quad V&= \frac{q}{C(\varphi )}, \end{aligned}$$

where $C(\varphi ): = \dfrac{d{\widehat{\rho }}}{d\varphi }(\varphi )$ denotes the capacitance.

• Charge-controlled memcapacitor:

3 $$\begin{aligned} \dot{\rho }&= q,\nonumber \\ \dot{q}&= I \quad \text {(input)},\nonumber \\ \text {(output)} \quad V&= K(\rho )q, \end{aligned}$$

where $K(\rho ):=\dfrac{d\widehat{\varphi }}{d\rho }(\rho )$ denotes the electrical elastance (inverse capacitance).

### Meminductor

An ideal meminductor^3^ is defined by a constitutive relationship between charge *q* and time-integrated flux-linkage $\sigma$, i.e.,

4 $$\begin{aligned} \sigma = \widehat{\sigma }(q) \ \text {or} \ q = \widehat{q}(\sigma ). \end{aligned}$$

Differentiating the latter with respect to time yields the following dynamical systems.

• Current-controlled meminductor:

5 $$\begin{aligned} \dot{q}&= \frac{\varphi }{L(q)},\nonumber \\ \dot{\varphi }&= V \quad \text {(input)},\nonumber \\ \text {(output)} \quad I&= \frac{\varphi }{L(q)}, \end{aligned}$$

where $L(q):=\dfrac{d\widehat{\sigma }}{dq}(q)$ denotes the inductance.

• Flux-controlled meminductor:

6 $$\begin{aligned} \dot{\sigma }&= \varphi ,\nonumber \\ \dot{\varphi }&= V \quad \text {(input)},\nonumber \\ \text {(output)} \quad I&= \varGamma (\sigma )\varphi , \end{aligned}$$

where $\varGamma (\sigma ):=\dfrac{d\widehat{q}}{d\sigma }(\sigma )$ denotes the inverse inductance.

## Dissipativity and cyclo-dissipativity theory

Let us first recall the basic definitions of dissipativity and passivity theory as originating from the seminal work of Willems^13^, see also Hill and Moylan^14^ and Van der Schaft^15^, and then turn attention to the less well-known, but thermodynamically very relevant, weaker notion of cyclo-dissipativity and cyclo-passivity.

Consider a system with state vector *x* and a vector of external (e.g., input and output) variables *w*. Consider a scalar-valued *supply rate* *s*(*w*). A function *S*(*x*) is said to be a *storage function* (with respect to the supply rate *s*) if along all trajectories of the system and for all $t_1 \le t_2$ and $x(t_1)$ it satisfies the *dissipation inequality*

7 $$\begin{aligned} S\big (x(t_2)\big ) - S\big (x(t_1)\big ) \le \int \limits _{t_1}^{t_2} s(w(t)) dt. \end{aligned}$$

Interpreting $s\big (w(t)\big )$ as ‘power’ supplied to the system at time *t*, and $S\big (x(t)\big )$ as stored ‘energy’ while at state *x*(*t*), this means that increase of the stored energy can only occur due to externally supplied power.

Following Willems^13^, the system is called *dissipative* (with respect to the supply rate *s*) if there exists a *non-negative* storage function *S*. [Since addition of an arbitrary constant to a storage function again leads to a storage function, the requirement of non-negativity of *S* can be relaxed to *S* being *bounded from below*.] Furthermore, it is called *lossless* (with respect to *s*) if there exists a non-negative storage function satisfying the dissipation inequality (7) with *equality*.

An external characterization of dissipativity, in terms of the behavior of the *w* trajectories, is the following^13^. Define for any *x* the expression (possibly infinite)

8 $$\begin{aligned} S_a(x):= \sup _{w, T\ge 0} - \int \limits _0^T s\big (w(t)\big ) dt, \end{aligned}$$

where the supremum is taken over all external trajectories $w(\cdot )$ of the system corresponding to initial condition $x(0)=x$, and all $T\ge 0$. Obviously, $S_a(x)\ge 0$. Then the system is dissipative with respect to the supply rate *s* if and only if $S_a(x)$ is *finite* for every *x*.

Interpreting as above *s*(*w*) as ‘power’ supplied to the system, $S_a(x)$ is the maximal ‘energy’ that can be extracted from the system at initial condition *x*, and the system is dissipative if and only if this maximal ‘energy’ is finite for any *x*. Furthermore, if $S_a(x)$ is finite for every *x* then $S_a$ is itself a non-negative storage function, and is in fact the *minimal* non-negative storage function (generally among many others).

Dropping the requirement of non-negativity of the storage function *S* leads to the notion of *cyclo-dissipativity*, respectively *cyclo-losslessness*^15–18^. First note that if a general, possibly indefinite, storage function *S* satisfies (7), then for all *cyclic* trajectories, i.e., such that $x(t_1)=x(t_2)$, we have that

9 $$\begin{aligned} \int \limits _{t_1}^{t_2} s\big (w(t)\big ) dt \ge 0, \end{aligned}$$

which holds with equality in case (7) holds with equality. This leads to the following external characterization of *cyclo*-dissipativity and *cyclo*-losslessness.

### Definition

A system is cyclo-dissipative if (9) holds for all $t_2\ge t_1$ and all external trajectories *w* such that $x(t_2)=x(t_1)$. In case (9) holds with equality, we speak about cyclo-losslessness. Furthermore, the system is called cyclo-dissipative with respect to $x^*$ if (9) holds for all $t_2\ge t_1$ and all external trajectories *w* such that $x(t_2)=x(t_1)=x^*$, and cyclo-lossless with respect to $x^*$ if this holds with equality.

Interpreting again *s*(*w*) as power provided to the system, cyclo-dissipativity thus means that for any cyclic trajectory the net amount of energy supplied to the system is non-negative, and zero in case of cyclo-losslessness.

The following theorem^17^ extends the results in Hill and Moylan^16^ and shows the equivalence between the external characterization of cyclo-dissipativity and cyclo-losslessness and the existence of storage functions.

### Theorem

*If there exists a function* *S* *satisfying the dissipation inequality* (7), *then the system is cyclo-dissipative, and it is cyclo-lossless if* *S* *satisfies* (7) *with equality. Conversely, assume the system is reachable from some ground-state* $x^*$*and controllable to this same state* $x^*$. [*It is immediate that this property is independent of the choice of* $x^*$.] *Define the (possibly extended) functions* $S_{ac}: \mathscr {X}\rightarrow \mathbb {R}\cup \infty$*and* $S_{rc} : \mathscr {X}\rightarrow - \infty \cup \mathbb {R}$*as*

10 $$\begin{aligned} S_{ac}(x)&= \mathop {\sup _{w, \, T\ge 0}}_{x(0)=x, \, x(T)=x^*} - \int \limits _0^Ts(w(t)) dt, \nonumber \\ S_{rc}(x)&= \mathop {\inf _{w, \, T\ge 0}}_{x(-T)=x^*, \, x(0)=x} \ \ \int \limits _{-T}^0s(w(t)) dt, \end{aligned}$$

*where the supremum and infimum are taken over all external trajectories* $w(\cdot )$*and* $T\ge 0$*such that* $x(0)=x,x(T)=x^*$, *respectively* $x(-T)=x^*,x(0)=x$. *Then the system is cyclo-dissipative with respect to* $x^*$*if and only if*

11 $$\begin{aligned} S_{ac}(x) \le S_{rc}(x), \ {for\;all} \ x \in \mathscr {X}\end{aligned}.$$

*Furthermore, if the system is cyclo-dissipative with respect to* $x^*$*then both* $S_{ac}$*and* $S_{rc}$*are storage functions, and thus the system is cyclo-dissipative. Finally*, $S_{ac}(x^*) = S_{rc}(x^*)=0$, *and any other storage function* *S* *satisfies*

$$\begin{aligned} S_{ac}(x) \le S(x) - S(x^*) \le S_{rc}(x), \ {for\;all} \ x \in \mathscr {X} \end{aligned},$$

*while if the system is cyclo-lossless* $S_{ac}(x)= S(x) - S(x^*)= S_{rc}(x)$, *implying uniqueness (up to a constant) of the storage function*.

Note that, unlike the dissipativity case, for a cyclo-dissipative system it may be possible to extract an *infinite* amount of energy from the system (since the storage function may not be bounded from below).

Finally, consider an input-state-output system

$$\begin{aligned} \dot{x}&= f(x,u),\\ y&= h(x,u), \end{aligned}$$

with state variables $x \in \mathscr {X}$, input(s) *u*, and output(s) *y*, with $w=(u,y)$. Then for any *differentiable* storage function *S* the dissipation inequality (7) is equivalent to its differential version^13–15^

$$\begin{aligned} \frac{\partial S}{\partial x}(x)f(x,u) \le s\big (u,h(x,u)\big ), \ {for\;all} \ x,u. \end{aligned}$$

In case of the *passivity* supply rate $s(w)=s(u,y)=y^Tu$, where $w=(u,y)$ and *u* and *y* are equally dimensioned vectors, the terminology ‘dissipativity’ in all of the above is replaced by the classical terminology of *passivity*, and cyclo-dissipativity by *cyclo-passivity*. In this case *u* and *y* typically are vectors of power-conjugate variables, like forces and velocities, and voltages and currents. For an input-affine system of form

$$\begin{aligned} \dot{x}&= f(x) + g(x)u,\\ y&= h(x), \end{aligned}$$

with state variables $x \in \mathscr {X}$, input(s) *u*, and output(s) *y*, cyclo-passivity amounts to the existence of a storage function (nonnegative in the case of passivity) $S:\mathscr {X}\rightarrow \mathbb {R}$ such that^14–16^

12 $$\begin{aligned} h(x) = g^T(x)\frac{\partial S}{\partial x}(x) \ \text {and} \ \left[ \frac{\partial S}{\partial x}(x)\right] ^T f(x) \le 0. \end{aligned}$$

This characterization of (cyclo-)passivity will be instrumental in analyzing the (cyclo-)passivity properties of the ideal memcapacitor and meminductor in the next section.

## Ideal energy-storing memelements are not cyclo-passive

Consider the charge-controlled memcapacitor (3). Suppose that $S(\rho ,q)$ is a storage function with respect to the passivity supply rate $s(I,V)=IV$. Then, according to (12), the system (3) is cyclo-passive if and only if the following two properties are satisfied:

13a $$\begin{aligned}&\frac{\partial S}{\partial q}(\rho ,q) = K(\rho ) q, \end{aligned}$$

13b $$\begin{aligned}&\frac{\partial S}{\partial \rho }(\rho ,q)q \le 0. \end{aligned}$$

From the equality (13a) it follows that $S(\rho ,q) = \frac{1}{2}K(\rho )q^2 + G(\rho )$, for some function $G(\rho )$. Substituting the latter into the inequality (13b) yields that

$$\begin{aligned} \frac{1}{2}K'(\rho )q^3 + G'(\rho )q \le 0, \end{aligned}$$

for all $\rho$ and *q*, and $(\cdot )'$ denotes the ordinary derivative with respect to the function’s argument. However, this readily implies that $G'(\rho )=0$ (and thus that *G* is a constant function) and $K'(\rho ) = \widehat{\varphi }''(\rho )=0$, which implies that $\varphi$ can at most be an affine function of $\rho$, i.e., $\varphi = K_0 + K_1\rho$. Hence the output equals $V=K_1 q$, which just constitutes an ordinary *linear* capacitor. In conclusion, a charge-controlled memcapacitor (3) is *not* cyclo-passive!

Performing the same analysis as above for the voltage-controlled memcapacitor (2), we see that $S(\varphi ,q)$ is a storage function if and only if

$$\begin{aligned} \frac{\partial S}{\partial q}(\varphi ,q) = \frac{q}{C(\varphi )} \ \Leftrightarrow \ S(\varphi ,q) = \frac{q^2}{2C(\varphi )}+G(\varphi ), \end{aligned}$$

and

$$\begin{aligned} \frac{\partial S}{\partial \varphi }(\varphi ,q)\dot{\varphi } \le 0 \ \Leftrightarrow \ \left[ -\frac{q^2}{2C^2(\varphi )}C'(\varphi ) + G'(\varphi ) \right] \frac{q}{C(\varphi )} = - \frac{C'(\varphi )}{2C^3(\varphi )}q^3 + \frac{G'(\varphi )}{C(\varphi )}q\le 0, \end{aligned}$$

which, using $q = C(\varphi )V$, implies that

14 $$\begin{aligned} \frac{1}{2}C'(\varphi )V^3 + G'(\varphi )V \le 0, \end{aligned}$$

for all $\varphi$ and *V*. Like before, the latter inequality can only be satisfied if and only if $C'(\varphi ) = G'(\varphi ) = 0$. This means that the only admissible constitutive relationship must be linear, i.e., $\rho = C_0 + C_1 \varphi$, which again implies an ordinary linear capacitor $q=C_1 V$, and *G* is again any constant function.

Thus, an ideal memcapacitor—either voltage- or charge-controlled—is not passive nor cyclo-passive! The same conclusion follows *mutatis-mutandis* for both the ideal current- and flux-controlled meminductors defined by (5) and (6), respectively. By the theory of “Dissipativity and cyclo-dissipativity theory”, this implies that for *any* nonlinear constitutive relationship in (1) or (4), there should *always* exist an input function (an ‘excitation profile’) that lets us extract more energy from the device than it is supplied with. An explicit construction of such an input function will be given in the next section, using parameters of a mechanical analogue; namely the mem-inerter. This construction can be directly converted to the explicit construction of an input function that extracts more energy than it supplies in case of ideal memcapacitors and meminductors.

## Overunity devices?

As mentioned in “Introduction”, a real-world example of an electrical device that unambiguously and closely resembles an ideal memcapacitor or an ideal meminductor satisfying (2) and (3) or (5) and (6), respectively, has not yet been found. The reason is that physical devices are necessarily cyclo-passive, while ideal memcapacitors and meminductors are not; as shown in “Ideal energy-storing memelements are not cyclo-passive”. This implies that there *exist* input functions that extract more energy than they need to supply during cyclic motions. In this Section, we will give an explicit construction of such a function. However, instead of using theoretically defined characteristics of ideal electrical energy-storing memelements from the literature^5,19^, or any arbitrary nonlinear constitutive relationship (1) or (4) for that matter, we prefer to use a more practically appealing mechanical device that by analogy exhibits exactly the same issues.

### Mechanical analogue: the mem-inerter

Adopting the Firestone (mobility) analogy^20^ for which voltage is considered analogous to velocity and current is analogous to force, suggests that the ideal voltage-controlled memcapacitor (2) can be considered as the electrical analogue of a recently proposed and experimentally realized mechanical displacement-dependent *mem-inerter*^21,22^. Such system exhibits a displacement-dependent inertance (mechanical ‘capacitance’) of the form

15 $$\begin{aligned} B(z) = b_0\left( \frac{w}{2}-z\right) , \end{aligned}$$

where $z \in [-w/2,w/2]$ denotes the relative displacement (mechanical ‘flux-linkage’) of the piston, and $b_0$ and *w* are the base inertance and the working width of the piston, respectively. Hence, we obtain the following dynamical system [compare with (2)]

16 $$\begin{aligned} \dot{z}&= \frac{p}{B(z)},\nonumber \\ \dot{p}&= F \quad \text {(input)},\nonumber \\ \text {(output)} \quad v&= \frac{p}{B(z)}, \end{aligned}$$

where *p* represent the linear momentum (mechanical ‘charge’), *F* the applied force (mechanical ‘current’), and $v=\dot{z}$ the associated velocity (mechanical ‘voltage’).

Alternatively, adopting the classical Maxwell–Kelvin analogy^20^ for which voltage is considered analogous to force and current is analogous to velocity, the mem-inerter (16) constitutes an equally valid mechanical analogue of an ideal current-controlled meminductor (5).

### Example of ‘free’ energy harvesting

Since the mem-inerter (16) is mathematically equivalent to the ideal voltage-controlled memcapacitor (2) and the ideal current-controlled meminductor (5), it is not passive nor cyclo-passive—as is shown in “Ideal energy-storing memelements are not cyclo-passive”. This implies that there exists an input signal for which we can extract more energy from the system than it is supplied with. Indeed, adopting the same parameters as in Zang et al.^21^ yields $b_0=9381.7$ [kg] and $w=0.1$ [m], and selecting for instance the following (zero-mean) periodic force profile (measured in newtons [N])

$$\begin{aligned} F(t) = 2.5\sin (\omega t) - 0.25\cos (\omega t) -5\sin (2\omega t) - 1.25\cos (2\omega t) + 2.75\cos (3\omega t) - 1.25\cos (4\omega t), \end{aligned}$$

with $\omega =0.5$ [rad/s] and $T=4\pi$ [s] the time of one full cycle.

**Figure 1:**
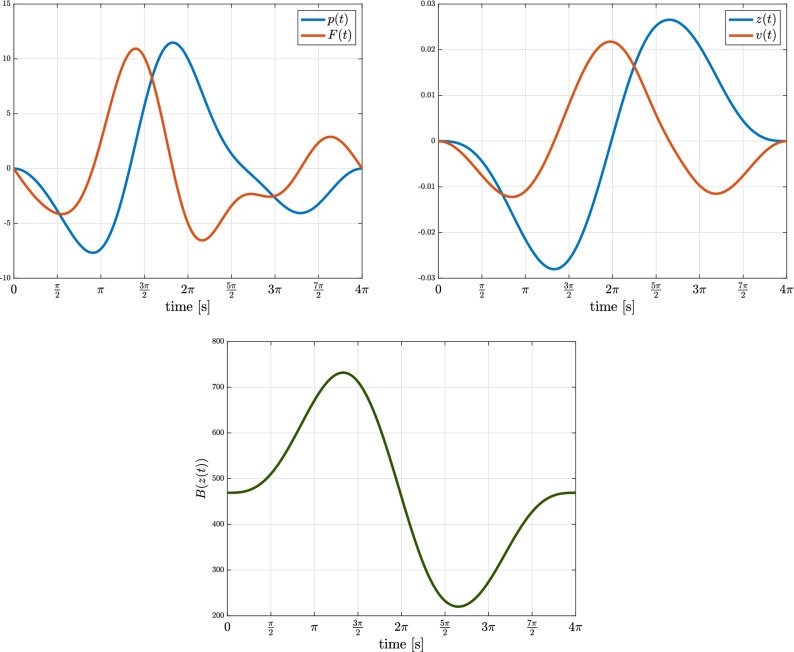
Input, state, and ouput trajectories (top) and inertance (bottom).

The simulation results are shown in Fig. 1. The initial conditions were set to zero and it is observed that both states start from and return to the same ground states, i.e.,

$$\begin{aligned} z(0) = z(T) = 0 \ \text {[m]} \ \text {and} \ p(0) = p(T) = 0 \ \text {[Ns]}. \end{aligned}$$

**Figure 2:**
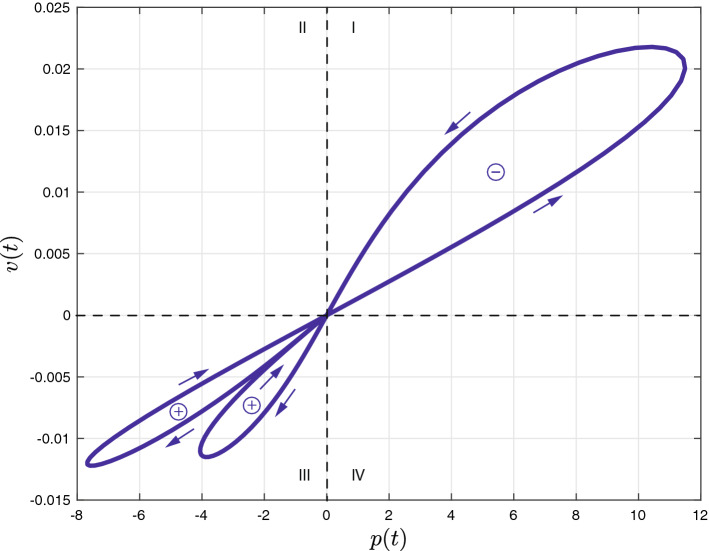
Lissajous plot of the velocity versus the momentum.

Interestingly, the supplied energy over one cycle equals

$$\begin{aligned} \oint \limits _0^{T} v(t)F(t)dt = \boxed {-0.056 \ \text {[J]}} \end{aligned}$$

which shows that there is more energy gained from the device than it is supplied with. Moreover, for each subsequent cycle *T*, the gained energy increases linearly with the number of cycles. This is also evident from the Lissajous plot in Fig. 2; the loop in quadrant I has a counter-clockwise orientation (energy gain), whereas the loops in quadrant III both have a clockwise orientation (energy lost); the energy gained clearly exceeds the loss of energy. Thus, we can apparently harvest ‘free’ energy from a mem-inerter, which confirms and exemplifies the overunity assertions of “Ideal energy-storing memelements are not cyclo-passive”.

## Concluding remarks

It is shown that ideal memcapacitors, ideal meminductors, and their analogies (like the recently proposed mem-inerter) constitute overunity systems. Overunity systems belong to the class of perpetual motion machines of the first kind and are so far non-existing devices in the real-world as they are in direct conflict with the First Law of thermodynamics^23^. For this reason alone, ideal real-world memcapacitors and meminductor will most probably never see the light of day.

Of course, one could argue that for a certain class of inputs an ideal memcapacitor or meminductor might exhibit a (cyclo-)passive behavior. Purely sinusoidal inputs, which are most often used in the literature to reveal pinched hystereses loops, are an example of periodic excitations that generally yield cyclo-passive or even cyclo-lossless input-output behavior. However, for a device to be truly (cyclo-)passive, such behavior should be observable for *all* possible excitation profiles.

On the other hand, one may ask if the ideal memcapacitor and meminductor models can be *rendered* cyclo-passive by adding small corrections or combining them with other ideal two-terminal devices, such as resistors, inductors, and capacitors^7^. As is clear from the analysis in “Ideal energy-storing memelements are not cyclo-passive”, in order to render an ideal voltage-controlled memcapacitor (cyclo-)passive, the left-hand side of the inequality (14) must be dominated. This can be accomplished by the addition of a conductance (inverse resistance), say $\gamma > 0$, resulting in

$$\begin{aligned} \frac{1}{2}C'(\varphi )V^3 + G'(\varphi )V \le \gamma V^2. \end{aligned}$$

However, it can be shown that generally a significant amount of conductance should be added. This, in turn, will also dominate the characteristics of the memcapacitor itself and obscure its effective working principles. Adding small corrections in the form of some extra parasitic capacitance or inductance will help neither since such additions do not alter the above inequality. The same reasoning applies to an ideal charge-controlled memcapacitor or a current- and flux-controlled meminductor.

In conclusion, ideal memcapacitors, ideal meminductors, and their possible analogies such as ideal mem-inerters are non-physical devices. Their realizability or their use as a realistic and reliable device model for mimicking real-world devices^3–5,9^ is therefore highly debatable.
